# Evaluation of glyceraldehyde-3-phosphate, prolylpeptidyl isomerase A, and a set of stably expressed genes as reference mRNAs in urate crystal inflammation

**DOI:** 10.1186/1756-0500-4-443

**Published:** 2011-10-25

**Authors:** Cristina Della Beffa, Frank Klawonn, Joseph P Menetski, H Ralph Schumacher, Frank Pessler

**Affiliations:** 1 Department of Cellular Proteomics, Helmholtz Centre for Infection Research, Inhoffenstr. 7, 38124 Braunschweig, Germany; 2 Department of Infection Genetics, Helmholtz Centre for Infection Research, Inhoffenstr. 7, 38124 Braunschweig, Germany; 3 Department of Epidemiology, Helmholtz Centre for Infection Research, Inhoffenstr. 7, 38124 Braunschweig, Germany; 4 Department of Computer Science, Ostfalia University of Applied Sciences, Salzdahlumer Str. 46/48, 38302 Wolfenbüttel, Germany; 5 Department of Immunology, Merck Research Laboratories, 126 East Lincoln Avenue Rahway, NJ 07065-4646, USA; 6 Division of Rheumatology, University of Pennsylvania, 3600 Spruce St, Philadelphia, PA 19104, USA; 7 Division of Rheumatology, Philadelphia VA Medical Center, University and Woodland Avenues, Philadelphia, PA 19104, USA; 8 University Children's Hospital, Faculty of Medicine "Carl-Gustav-Carus", Technical University Dresden, Fetscherstr. 74, 01307 Dresden, Germany

## Abstract

**Background:**

The murine air pouch membrane represents an easily accessible tissue for studies on gene regulation in acute inflammation. Considering that acute inflammation may affect expression of molecular reference genes, we evaluated the expression of glyceraldehyde-3-phosphate dehydrogenase (GAPDH) and prolylpeptidyl isomerase A (PPIA) in the air pouch membrane during a complete time course of urate crystal inflammation and correlated the results with expression of interleukin (IL)-1β and hypoxia inducible factor (HIF)-1α. In addition, we aimed to identify alternate potential reference genes.

**Methods:**

Using custom microfluidic real-time PCR arrays, the expression of 96 genes including GAPDH, PPIA, IL-1β, and HIF-1α was determined in dissected air pouch membranes 1, 4, 9, 18, 27, and 50 hours (h) after injecting monosodium urate (MSU) crystals into the pouch. One-way ANOVA was used to detect differential gene expression throughout the time course. Using the genes on these arrays as a convenience sample, alternate candidate reference genes were sought (1) with a biostatistical approach and (2) using the geNorm software tool.

**Results:**

Pouch leukocytes peaked at t = 9h and declined toward t = 50h. PPIA expression was not differentially regulated (p = 0.52, ANOVA). In contrast, GAPDH mRNA increased steadily after crystal injection, reaching a maximal 2.8-fold increase at t = 18h (p = 0.0006, *t *test), which followed a marked induction of IL-1β (max., 208-fold at t = 4h, p = 8.4 × 10^-5^, *t *test) and HIF-1α (max., 6.6-fold at t = 4h, p = 0.00025, *t *test). Fifteen genes were artifactually identified as "significantly regulated" when Ct values were normalized against GAPDH expression. The biostatistical approach and the geNorm analysis identified overlapping sets of candidate reference genes. Both ranked PPIA as the best candidate, followed by defender against cell death 1 (DAD1) and high-mobility group B1 (HMGB1).

**Conclusions:**

GAPDH mRNA expression is up-regulated in urate crystal inflammation, possibly due to inflammation-associated hypoxia. Using GAPDH mRNA for molecular normalization resulted in significant artifacts in the calculated expression of the target mRNAs. PPIA and other stably expressed genes promise to be more appropriate reference genes in this model.

## Background

### Glyceraldehyde-3-phosphate dehydrogenase (GAPDH) and prolylpeptidyl isomerase A (PPIA) as potential reference genes

GAPDH is often used for molecular normalization of gene expression data from microarrays or real-time reverse transcriptase polymerase chain reactions (qPCR). This is based on the assumption that expression of this "housekeeping gene" does not change much during the life cycle of most cells and can thus be used as a relatively constant reference signal. However, while this notion has been substantiated in some scenarios, there are clear examples that GAPDH mRNA expression can vary (e.g., [1-3]). Notably, hypoxia can induce GAPDH mRNA levels, likely because binding of a complex of the inducible α subunit and the constitutively expressed β subunit of hypoxia inducible factor (HIF)-1 to a hypoxia response element (HRE) in the GAPDH promoter region can increase transcription of this gene [4-6]. Considering that hypoxia is a well documented feature of inflammatory cells and inflamed tissues [7,8], including the synovial membrane [9], that HIF-1 can be activated in inflammation due to toll-like receptor (TLR) signaling [8,10], and that HIF-1α is expressed widely in inflamed synovial membranes [11], GAPDH may not be a suitable reference gene for molecular normalization in gene expression studies of acute synovitis, including crystal inflammation.

PPIA (also known as cyclophilin A) is a ubiquitously expressed intermediate factor of calcium/calmodulin signaling. Its activity is regulated predominantly at the post-transcriptional level. While it has been validated as a useful reference gene for qPCR in specific scenarios [12,13], its transcriptional regulation has been demonstrated in hypoxic cells [4] and in at least one example of a chronically inflamed tissue [14]. Thus, there is reason to suspect that it, too, may be a suboptimal reference gene in studies on inflammation.

### The murine air pouch model of inflammation

The murine air pouch is a bursa-like structure that is lined with a membrane resembling the lining of human joints, both histologically and biochemically [15]. The pouch lumen is an easily accessible space, and different inflammatory processes can be elicited easily by injecting the respective pro-inflammatory agent. The air pouch membrane can be removed from the mouse nearly quantitatively by blunt dissection and thus provides an attractive system for studying inflammation-related gene expression changes in a synovium-like tissue, while minimizing transcriptional noise hailing from the adjacent structures [16]. However, the usefulness of common reference genes for real-time PCR analysis of this tissue has not been examined. Considering the fulminant inflammatory reaction that ensues after injecting the crystals, changes in basic elements of cellular metabolism affecting expression of otherwise stably expressed genes appear likely. Using a time course experiment spanning initiation, peak and resolution of inflammation in the air pouch, we have therefore evaluated GAPDH and PPIA as reference genes for molecular normalization in this model. We find relatively stable expression of PPIA, but a steady rise of GAPDH mRNA levels that peak after maximal leukocyte accumulation in the pouch lumen, and we speculate that inflammation-associated hypoxia and/or oxidative stress are causative factors.

## Methods

### Murine air pouch model

**Figure 1:**
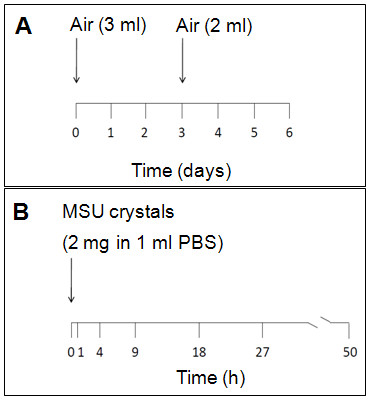
**Schematic timelines of the formation of the air pouch (A) and the time course experiment (B)**. **A**. Three ml of filtered air are injected under the dorsal skin. The resulting air pouch is reinflated on day 3 with an additional injection of 2 ml. A newly formed thin membrane lines the pouch lumen by day 6. **B**. Two mg endotoxin-free MSU crystals are injected into the pouch on day 6. At the indicated time points (x-axis), the inside of the pouch is lavaged with PBS to determine the number of leukocytes, and the pouch membrane is then isolated from the surrounding tissues by blunt dissection and processed for gene expression analysis. Four mice were used at t = 1h and t = 4h, and 3 mice at each of the other time points.

A 50-hour time course of urate crystal inflammation was carried out in the murine air pouch as described [16,17] (Figure 1). Briefly, air pouches were raised on the backs of 6-week-old female BALB/c mice by subcutaneously injecting 3 ml sterile air and reinflating with 2 ml sterile air on day 3. Endotoxin-free monosodium urate crystals were synthesized according to the McCarty method [18]. A suspension of 2 mg of crystals in 1 ml endotoxin-free phosphate buffered saline was injected into 6-day-old pouches. Mice (3-4 per time point) were killed 1, 4, 9, 18, 27, and 50 hours (h) after injecting the crystals, exudate leukocytes were quantified with a hemocytometer, and pouch membranes obtained by blunt dissection as described [16]. The t = 0h membranes were obtained at the beginning of the experiment from air pouches which had not been injected with crystals or buffer. The study was approved by the Institutional Animal Care and Research Committee of the Philadelphia VA Medical Center.

### Quantitative PCR analysis

Membranes were homogenized, total RNA extracted with RNeasy spin columns (Qiagen, Valencia, CA), and cDNA synthesized with Superscript II reverse transcriptase (Invitrogen, Carlsbad, CA), following the recommendations of the manufacturers. Expression of 96 target genes was quantified on an HT7900 Sequence Analyzer (Applied Biosystems [ABI], Foster City, CA) using custom microfluidic Taqman PCR arrays (ABI) following the manufacturer's protocols (http://www.appliedbiosystems.com). The arrays had been custom designed to contain three reference genes (18S rRNA, PPIA, and GAPDH) and 93 target genes relating to several functional pathways and processes (cell cycle, apoptosis, prostaglandin metabolism, inflammasome components, cytokines, defensins, matrix metalloproteinases, markers for specific cell types, and several miscellaneous targets of interest) to study their regulation, if any, in urate crystal inflammation. While the present manuscript focuses on reference gene selection, the analysis of expression of the functional pathways will be the subject of a separate publication.

### Pre-analytic data processing

Ten of the 96 genes on the arrays were removed from the analysis because two or more values were missing and this lack of detection could not be explained by a biologically plausible absence of expression at beginning or end of the time course. A Ct of 40 was assigned for missing values when absence of detection was likely due to physiological lack of expression at beginning or end of the time course; the mean Ct value of the time point was used when missing values were likely due to technical failure. All Ct values were subtracted from 40 such that higher Ct values corresponded to higher gene expression. To avoid the value 0 for samples with Ct = 40, the Laplace correction (adding a pseudocount of 1 to all values of the data set) [19] was used, thus obtaining (41-Ct). 18S rRNA, which is routinely included on these custom PCR arrays by the manufacturer, had to be excluded from the analysis because a batch effect was noted in principal component analysis (results not shown), which was due to low detection levels for this gene on three of the arrays. The Ct values of 18S rRNA did not correlate with mean Ct values across the arrays. Therefore, they did not reflect low overall gene expression in the affected samples, but were likely due to a manufacturing error. Eighty-five of the original 96 genes thus passed the preanalytic quality screen and were included in the analyses.

### Statistical analyses

Differential regulation in the time course of target genes of interest was determined with one-way ANOVA and the Kruskal-Wallis test, comparing t = 0h against the subsequent time points. P values were adjusted for multiple testing using the Benjamini-Hochberg (BH) correction method. Similar results were obtained with the two methods, but only p values obtained with ANOVA are shown for the sake of simplicity. For each target, a *t *test was performed between t = 0h and the time point associated with the greatest change in expression. Variation in gene expression was also assessed by median absolute deviation from the median (MAD) [20]. MAD is a more robust measure of data variability than standard deviation, which is obtained by multiplying MAD by a constant scaling factor. All statistical analyses were performed using code written in the R programming language (R project for statistical computing; http://www.r-project.org). The software tool geNorm [21] (available at http://www.biogazelle.com/genormplus/genorm) was also used to identify potential reference genes. It ranks a group of candidate reference genes according to the M value, i.e. the mean of all the standard deviation values between the Ct values of the gene in question and the Ct values of each of the other potential reference genes under consideration. A lower M value denotes a higher rank among the candidates.

## Results

### Up-regulation of GAPDH, but not PPIA, mRNA in urate crystal inflammation

**Figure 2:**
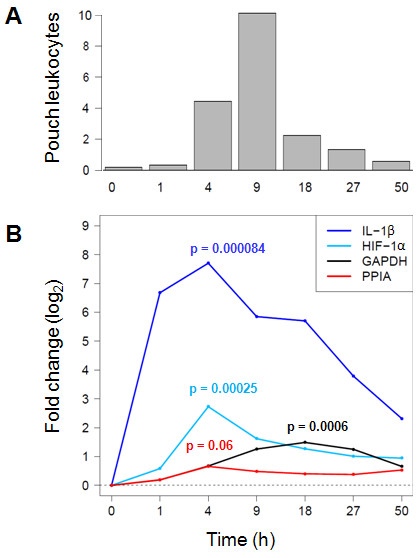
**Expression of IL-1β, HIF-1α, GAPDH, and PPIA in the air pouch membrane in a 50-hour time course of MSU crystal inflammation**. **A**. Leukocyte numbers were determined in lavaged pouch exudates by manual cell counting with a hemocytometer. Values are expressed as 1 × 10^6 ^leukocytes/pouch (y-axis). **B**. Changes in expression of IL-1β, HIF-1α, GAPDH and PPIA relative to t = 0h. Four mice were used at t = 1h and t = 4h, and 3 mice at each of the other time points. For each target, the p value (*t *test) is indicated at the time point of maximal fold change with respect to t = 0h.

Quantification of pouch exudate leukocyte counts, the gold standard for measuring inflammation in this model, revealed a rapid evolution of inflammation that peaked at t = 9h and then returned to near normal by t = 50h (Figure 2A). A dramatic surge in IL-1β mRNA level in the membrane preceded this peak of leukocyte transmigration by several hours, reaching a 208-fold increase at t = 4h. HIF-1α was also up-regulated significantly (p = 0.000067, ANOVA), albeit less dramatically (maximal increase, 6.6-fold at t = 4h, p = 0.00025, *t *test). Inspection of the relative expression values of PPIA revealed an apparent mild increase after t = 0h. Even though the apparent difference at the maximum (t = 4h) with respect to t = 0h approached significance (p = 0.06, *t *test), PPIA expression throughout the entire time course was not regulated significantly (p = 0.52, ANOVA) (Figure 2B). In contrast, GAPDH mRNA levels increased 4h after crystal injection, peaked at t = 18h, and began to decline thereafter (p = 0.023, ANOVA). The differences compared with t = 0h were most significant at t = 18h (2.8-fold change, p = 0.0006, *t *test). Thus, GAPDH expression was up-regulated significantly after the peak of inflammation in the pouch membrane. In contrast, PPIA was expressed relatively stably.

To exclude that the rise in GAPDH mRNA merely reflected a general induction of transcription at these time points or systematic differences in RNA or cDNA amount or quality, the mean Ct value across all 85 targets and samples was calculated for each time point. This overall mean transcription peaked at t = 4h and thus clearly followed different kinetics than regulation of GAPDH.

### Artifacts resulting from normalization against GAPDH expression

**Figure 3:**
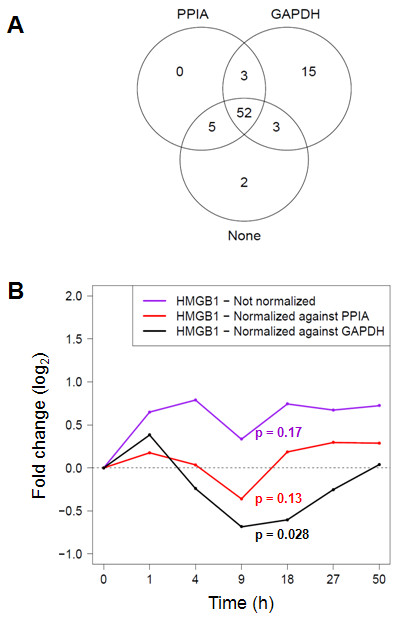
**Comparison of normalization strategies**. **A**. Venn diagram demonstrating differences in the populations of regulated mRNAs that result when the Ct values from the PCR arrays were not normalized ("None") or normalized against PPIA or GAPDH expression. The analysis is based on 85 of the 96 targets on the PCR arrays which passed an initial quality screen for missing values (see Methods). The intersecting lines define shared and distinct target gene populations. The values refer to the number of genes apparently regulated significantly (fold change ≥ 1.5, p < 0.05) in the time course. The three methods identified the following numbers of significantly regulated genes: no normalization, 62; PPIA normalization, 60; and GAPDH normalization, 73. **B**. Artifactual down-regulation of HMGB1 mRNA due to normalization against GAPDH. Expression of HMGB1 throughout the time course was determined in the non-normalized (violet line), GAPDH-normalized (black line), and PPIA-normalized (red line) data sets. P values for significance of regulation were determined with one-way ANOVA: no normalization, 0.52; PPIA normalization, 0.074; GAPDH normalization, 0.0037. Fold change with respect to t = 0h is plotted on the y-axis. For each normalization condition, the p value (*t *test) is indicated at the time point of maximal fold change.

The rise in GAPDH expression suggested that it should not be used as a reference mRNA in this model. To assess the magnitude of artifacts resulting from molecular normalization to GAPDH in this data set, the number of significantly regulated (fold change ≥ 1.5, p < 0.05 by ANOVA with BH correction for multiple testing) target genes was computed under three conditions: (1) without prior normalization, (2) normalized against GAPDH, and (3) normalized against PPIA (Figure 3A). Whereas there was good agreement between the non-normalized and the PPIA-normalized data sets, normalization against GAPDH resulted in the identification of 15 unique "regulated" mRNAs. To exemplify one specific artifact resulting from normalizing against GAPDH, the kinetics of the expression of HMGB1, which was identified as "regulated" only in the GAPDH-normalized data set, were then plotted under each of the three normalization conditions (Figure 3B). Whereas similar kinetics resulted in the non-normalized and PPIA-normalized data sets, GAPDH normalization led to an apparent down-regulation of HMGB1 mRNA (p = 0.0037, ANOVA) which correlated inversely with GAPDH up-regulation. Thus, using GAPDH as a molecular reference led to significant changes in data interpretation in this model, whereas PPIA-normalized values agreed better with the data obtained from the non-normalized data set.

### Identification of alternate candidate reference mRNAs

**Figure 4:**
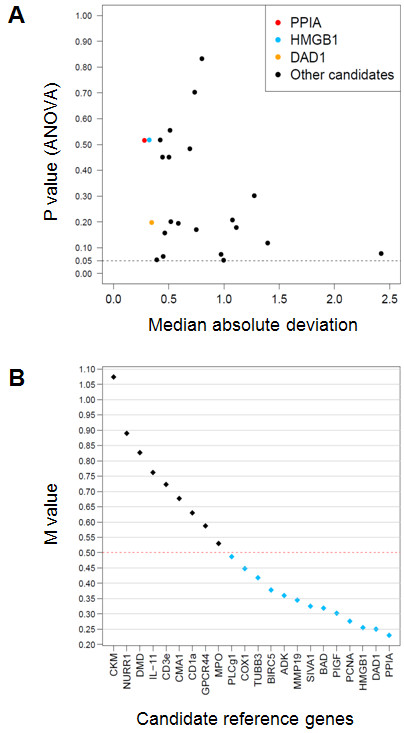
**Identification of candidate reference genes**. **A**. Median absolute deviation from the median (MAD) of 23 genes which were identified with ANOVA as "not regulated" (p ≥ 0.05) is plotted against the p values determined by ANOVA. Genes possessing low MAD but high p values (p >> 0.05) would constitute candidate reference genes. The three genes with the lowest MAD are identified by red (PPIA), orange (DAD1) and light blue (HMGB1) dots. The dotted line represents the p value threshold of 0.05. **B**. geNorm analysis of the same 23 genes as in A. The final output contains 22 genes since geNorm excluded IFN-γ due to excess missing values. The y-axis represents the average expression stability (M value) of the remaining 22 genes. PPIA, DAD1 and HMGB1 have the lowest M values and constitute the best candidate reference genes. Abbreviations: ADK, adenosine kinase; BAD, Bcl associated death promoter; BIRC5, baculoviral IAP repeat containing 5; CKM, creatine kinase (muscle); CD3e, cluster of differentiation 3, ε subunit; CD1a, cluster of differentiation 1a; CMA1, mast cell chimase; COX1, cyclooxygenase 1; DAD1, defender against cell death 1; DMD, dystrophin; GPCR44, G-protein coupled receptor 44; HMGB1, high mobility group B1; IL-11, interleukin 11; MMP19, matrix metalloproteinase 19; MPO, myeloperoxidase; NURR1, nuclear receptor related 1; PCNA, proliferating cell nuclear antigen; PIGF, phosphatidyl inositol glycan anchor biosynthesis, class F; PLCg1, phospholipase C γ1; PPIA, prolylpeptidyl isomerase A; SIVA1, SIVA1 apopotosis-inducing factor; TUBB3, tubulin β3.

Among the 85 genes on the arrays that passed the initial quality screen (see Methods), one-way ANOVA identified 23 genes as "not regulated" (p ≥ 0.05, after BH correction, using the non-normalized data set). These were then used as a convenience sample to screen for additional candidate reference genes. Figure 4A represents, for each target mRNA in all 23 samples, a plot of variation in Ct values, defined by MAD, versus the p value obtained with one-way ANOVA. PPIA, HMGB1, and DAD1 were the three mRNAs with the lowest MAD values (0.28, 0.32 and 0.34, respectively), and all three also had high p values and thus constituted the best candidates.

In an independent approach, the same set of 23 non-regulated genes was analyzed with the software program geNorm. Thirteen genes had M values < 0.5, a generally accepted cut-off for the identification of potentially useful reference genes [21] (Figure 4B). Of note, this unbiased screen identified the same top three genes as the MAD/ANOVA approach, PPIA (M = 0.23) being the best candidate, followed by DAD1 (M = 0.25) and HMGB1 (M = 0.26).

**Figure 5:**
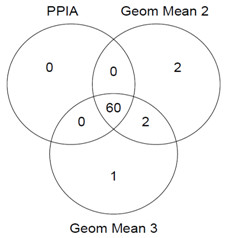
**Normalization against geometric means**. Venn diagram demonstrating differences in the populations of mRNAs identified as "regulated" when the Ct values from the PCR arrays were normalized against PPIA alone, against the geometric mean of PPIA and DAD1 ("Geom Mean 2") or the geometric mean of PPIA, DAD1 and HMGB1 ("Geom Mean 3"). The analysis is based on the same 85 targets as in Figure 3A. The intersecting lines define shared and distinct target gene populations. The values refer to the number of genes apparently regulated significantly (fold change ≥ 1.5, p < 0.05) in the time course. The three methods identified the following numbers of significantly regulated genes: PPIA alone, 60; Geom Mean 2, 64; Geom Mean 3, 63.

To test whether combinations of these three genes would result in further improvement in normalization, the Ct values of the genes on the arrays were normalized against the geometric mean of the Ct values of PPIA, DAD1 and HMGB1. Compared with normaliziation against PPIA alone, 4 additional regulated genes were identified when the geometric mean of PPIA and DAD1 was used, and 3 when the geometric mean of all three genes was used (Figure 5).

## Discussion

### Inutility of GAPDH as reference gene in MSU crystal inflammation

We detected significant up-regulation of GAPDH expression in the anticlimactic phase of acute urate crystal inflammation in this simple tissue resembling the human synovial membrane. These results suggest strongly that GAPDH should not be used as a reference gene in urate crystal inflammation and, likely, similar forms of acute inflammation driven by innate immunity. It remains to be determined whether this also applies to chronic or less fulminant inflammatory processes, but caution is indicated. In any case, these results underscore how important it is to test for any systematic regulation of GAPDH mRNA expression before considering it for molecular normalization in a new experimental system.

### Potential mechanisms of GAPDH induction in MSU crystal inflammation

What might be the underlying mechanism that increased transcription of this enzyme? The presented data are the first report of HIF-1α induction in urate crystal inflammation, but the available evidence from related inflammatory systems allows us to speculate that the increased GAPDH expression is due to increased HIF-1α activity. This might result from at least two synergizing processes, (1) higher HIF-1α protein levels due to increased stability resulting from proline hydroxylation (which could not be assessed in our mRNA-based analysis), and (2) increased transcription of the HIF-1α gene within the first hours of crystal injection (Figure 2). These changes may arise from activation of more than one molecular pathway. Urate crystals induce fulminant inflammation in the air pouch that is characterized by massive induction of innate immunity. The crystals are sensed as danger-associated molecular patterns (DAMPS) by the NALP3 inflammasome (leading to the proteolytic processing and release of IL-1β), and by other receptors including TLR 2 and 4 [22]. Notably, TLR 2 and 4 (as well as TLR 7 and 8, but these are not known to be activated by urate crystals) have been shown to activate HIF-1α activity [8,10]. This can be seen as part of the intracellular adaptation to hypoxic and oxidative stress that infiltrating inflammatory cells are invariably exposed to. Injecting urate crystals into the air pouch causes a major influx of innate immune cells, consisting of neutrophilic granulocytes, monocytes (which express TLR 2 and 4), and mast cells [23] (whose activation also leads to increased HIF-1α activity [24]), all of which ultimately results in the release of major proinflammatory cytokines and reactive oxygen species (ROS). Moreover, focal hypoxia is a common feature of inflamed tissues [7], including the synovial membrane [9], likely due to increased oxygen demand in the tissue in the presence of vascular congestion. A recent report in this journal on expression of GAPDH in tumor and non-tumor cell lines clearly demonstrated that GAPDH expression increases under hypoxic conditions in a cell-type specific manner [2], likely due to HIF-1α induction. GAPDH is a key enzyme of glycolysis. A HIF-1α-induced increase in GAPDH activity would activate this pathway, thus improving the intracellular energy balance by providing additional ATP molecules. In addition, intracellular ROS can induce HIF-1α activity [8]. Besides these intracellular ROS, which serve as signaling molecules in innate immune cells during activation, neutrophil and mast cell degranulation leads to additional oxidative stress in the tissue, and increased GAPDH activity may increase survival of affected cells by increasing the availability of ATP and reducing equivalents. Thus, the observed induction of GAPDH in the presented study may be explained biochemically in two, not mutually exclusive, ways: (1) as an inevitable result of TLR signal transduction triggered by urate crystals and (2) as a central feature of the tissue adaptation to hypoxia and oxidative stress. Future studies should be directed at testing these models experimentally.

### PPIA, DAD1 and HMGB1 as potential reference genes for urate crystal inflammation

The MAD and geNorm analyses highlighted several stably expressed genes, and both of these unbiased approaches identified PPIA as the best candidate reference gene. PPIA has been validated for molecular normalization in qPCR analysis of, for instance, colorectal and breast cancer tissue [12,13]. It now promises to be a useful reference gene also in urate crystal inflammation and, possibly, similar states of inflammation. DAD1 is a subunit of the enzyme oligosaccharyltransferase and can counteract apoptotic cell death [25]. Interestingly, MSU crystals inhibit neutrophil apoptosis [26]. While it is not known whether this process involves DAD1 activity, our results indicate that the crystals at least do not affect transcription of the DAD1 gene. HMGB1 is a multifunctional protein that usually resides in the nucleus and functions in DNA repair [27]. But it also plays important extracellular roles in innate immunity as an "alarmin", where it is released from cells through activation of the very NALP3 inflammasome that is activated by MSU crystals [28]. Although this has not been addressed experimentally, induction of HMGB1 activity in response to MSU crystal is highly likely. Its identification as a stably expressed gene in MSU crystal inflammation was, therefore, unexpected. A likely explanation is that its activity is regulated predominantly, if not exclusively, at the post-transcriptional level in this type of acute but self-limiting process.

Using the geometric mean of a combination of two or more reference genes has become a commonly recommended strategy for molecular normalization of RT-PCR data sets [29]. The geNorm analysis suggested that the combination of PPIA, DAD1 and HMGB1 would constitute the optimal normalization strategy in the current data set. Using this combination affected the number of genes identified as "significantly regulated" in a relatively minor way, which might be viewed as "fine tuning" of normalization against PPIA alone. Additional changes due to using this combination may be more subtle and, for instance, affect the measured kinetics of some of the regulated genes.

### Limitations

This study is limited by the fact that reference genes that are commonly used in inflammation-related gene expression studies, notably β-actin and 18S and 28S rRNA, were not included in the analysis and that no attempt was made to elucidate the mechanisms underlying GAPDH induction or to verify expression of the protein products of the studied mRNAs. Moreover, DAD1 and HMGB1 and the other stably expressed genes other than PPIA were included on the arrays *a priori *not as potential reference genes but in order to test whether their expression might be functionally regulated in urate crystal inflammation. However, our results do suggest that a *post hoc *data mining approach of PCR arrays may be useful to screen for additional candidate reference genes. Clearly, future studies of urate crystal inflammation should be designed to (1) further validate PPIA, DAD1 and HMGB1 as reference genes and to compare their performance directly with that of commonly used reference genes and (2) to provide more experimental evidence for the hypothetical model of HIF-1α-associated GAPDH induction outlined at the beginning of this section.

## Conclusions

GAPDH mRNA expression is up-regulated in urate crystal inflammation, possibly due to inflammation-associated hypoxia and/or oxidative stress. Using GAPDH mRNA for molecular normalization resulted in significant artifacts in the calculated expression of the target mRNAs, and it should therefore not be used as a reference mRNA in studies on MSU crystal inflammation. PPIA and other stably expressed genes, including DAD1 and HMGB1, promise to be more appropriate reference genes in this model.

## Competing interests

The authors declare that they have no competing interests.

## Authors' contributions

CDB and FK analyzed the data and participated in writing the manuscript. JPM provided laboratory space and equipment, helped with data analysis, and edited the manuscript. HRS provided laboratory resources and MSU crystals, participated in study design and data analysis, and edited the manuscript. FP designed and oversaw the study, provided animal care and laboratory work, and wrote the manuscript. He had access to all data and takes responsibility for their integrity. All authors read and approved the final manuscript.
